# Prediction of linear B-cell epitopes of hepatitis C virus for vaccine development

**DOI:** 10.1186/1755-8794-8-S4-S3

**Published:** 2015-12-09

**Authors:** Wen-Lin Huang, Ming-Ju Tsai, Kai-Ti Hsu, Jyun-Rong Wang, Yi-Hsiung Chen, Shinn-Ying Ho

**Affiliations:** 1 Department and Institute of Industrial Engineering and Management, Minghsin University of Science and Technology, Xinfeng Hsinchu, Taiwan; 2 Institute of Bioinformatics and Systems Biology, National Chiao Tung University, Hsinchu, Taiwan; 3 Department of Biological Science and Technology, National Chiao Tung University, Hsinchu, Taiwan

## Abstract

**Background:**

High genetic heterogeneity in the hepatitis C virus (HCV) is the major challenge of the development of an effective vaccine. Existing studies for developing HCV vaccines have mainly focused on T-cell immune response. However, identification of linear B-cell epitopes that can stimulate B-cell response is one of the major tasks of peptide-based vaccine development. Owing to the variability in B-cell epitope length, the prediction of B-cell epitopes is much more complex than that of T-cell epitopes. Furthermore, the motifs of linear B-cell epitopes in different pathogens are quite different (e. g. HCV and hepatitis B virus). To cope with this challenge, this work aims to propose an HCV-customized sequence-based prediction method to identify B-cell epitopes of HCV.

**Results:**

This work establishes an experimentally verified dataset comprising the B-cell response of HCV dataset consisting of 774 linear B-cell epitopes and 774 non B-cell epitopes from the Immune Epitope Database. An interpretable rule mining system of B-cell epitopes (IRMS-BE) is proposed to select informative physicochemical properties (PCPs) and then extracts several if-then rule-based knowledge for identifying B-cell epitopes. A web server Bcell-HCV was implemented using an SVM with the 34 informative PCPs, which achieved a training accuracy of 79.7% and test accuracy of 70.7% better than the SVM-based methods for identifying B-cell epitopes of HCV and the two general-purpose methods. This work performs advanced analysis of the 34 informative properties, and the results indicate that the most effective property is the alpha-helix structure of epitopes, which influences the connection between host cells and the E2 proteins of HCV. Furthermore, 12 interpretable rules are acquired from top-five PCPs and achieve a sensitivity of 75.6% and specificity of 71.3%. Finally, a conserved promising vaccine candidate, PDREMVLYQE, is identified for inclusion in a vaccine against HCV.

**Conclusions:**

This work proposes an interpretable rule mining system IRMS-BE for extracting interpretable rules using informative physicochemical properties and a web server Bcell-HCV for predicting linear B-cell epitopes of HCV. IRMS-BE may also apply to predict B-cell epitopes for other viruses, which benefits the improvement of vaccines development of these viruses without significant modification. Bcell-HCV is useful for identifying B-cell epitopes of HCV antigen to help vaccine development, which is available at http://e045.life.nctu.edu.tw/BcellHCV.

## Background

Infection with the hepatitis C virus (HCV) often results in chronic hepatitis, liver cirrhosis, and hepatocellular carcinoma [1]. HCV presents high genetic heterogeneity [2], and HCV species are currently classified into 11 genotypes with 80 subtypes within each genotype [3]. Therefore, no vaccine is currently available [4]; however, some therapies have proven effective against some, but not all, genotypes [5]. HCV is an enveloped virus with two types of surface glycol-proteins, E1, and E2. The two types of glycoprotein epitopes are targets for the neutralization of antibody responses [6,7]. Some recent approaches to vaccine development have focused on HCV envelope structures [5,6,8].

Previously, the development of HCV vaccines has mainly focused on T-cell immune response [4,9-12]. Prabdial-Sing et al. performed sequence-based *in silico *analysis of HCV epitopes using algorithms to predict the immunogenicity of their variants from other less studied genotypes [13]. Li, et al. find that the two HLA epitopes may contribute to design the HCV vaccine for the Chinese population [4] and Aqsa, et al. report that the glycoprotein 2 of HCV-3a is an ideal target for vaccine design [10]. Despite identifying linear B-cell epitopes that can stimulate B-cell response, is one of the major tasks to design peptide-based vaccine; there are only few researches to analyze the B-cell immune response of HCV. Furthermore, design a predictor for B-cell epitopes, which have high variable epitope length, is more complex than predictor for T-cell epitopes [14].

**Figure 1:**
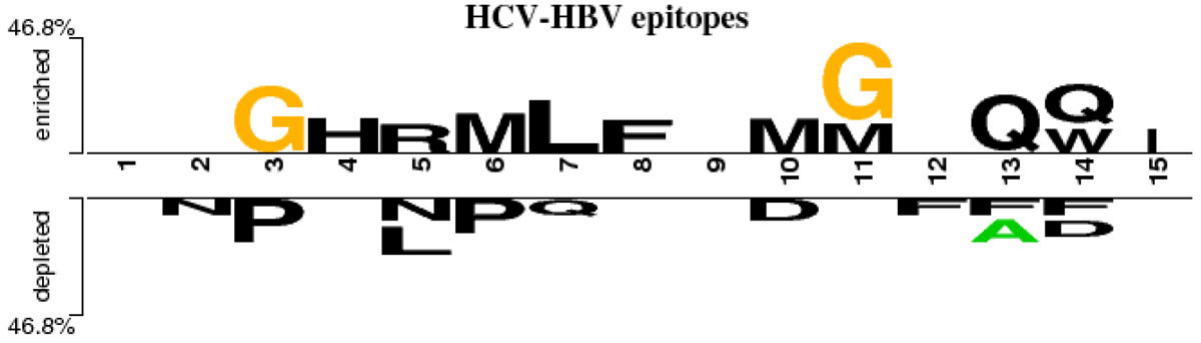
**Sequence logo of linear B-cell epitopes in hepatitis C virus and hepatitis B virus**. The sequence logo is generated using Two Sample Logo tool [48] with p-value < 0.05 criterion. The upper and lower motifs are the B-cell epitopes of hepatitis C virus (HCV) and hepatitis B virus (HBV), respectively.

On the other hand, some alternative computational methods (Table 1) have been developed for prediction of linear B-cell epitopes. These prediction methods mainly focus on peptides of a fixed length and use these peptides as an input to various machine learning models, including the Markov model (HMM), the artificial neural network (ANN), the support vector machine (SVM) [14-19]. However, the underperformances of these general-purpose methods [20,21] and the significantly differenent sequence context of HCV from the hepatitis B virus counterpart (Figure 1) motivate this work to develop a specific method/tool for identifying B-cell epitopes of HCV.

**Table 1:** Representative peptide-based methods for predicting linear B-cell epitopes.

Method	Number of propensities	Number of features	Propensity/features	Classifier
ABCpred [12]	single	400	AAP propensity	Neural network (NN)
BCPred [15]	single		AAP propensity	SVM
BepiPred [14]	single		Hydrophilicity propensity	Hidden Markov Model (HMM)
GFSMLP [17]	8	160	Solvent accessibility, and beta-turn propensities	SVM, NN
BayesB [18]	None	400	Position-specific scoring matrix (PSSM) with PSI-BLAST	SVM
SVMTriP [19]	single	8000	Tri-peptide	SVM
Bcell-HCV (this work)	6 [49]	34	PCP features	SVM

**Figure 2:**
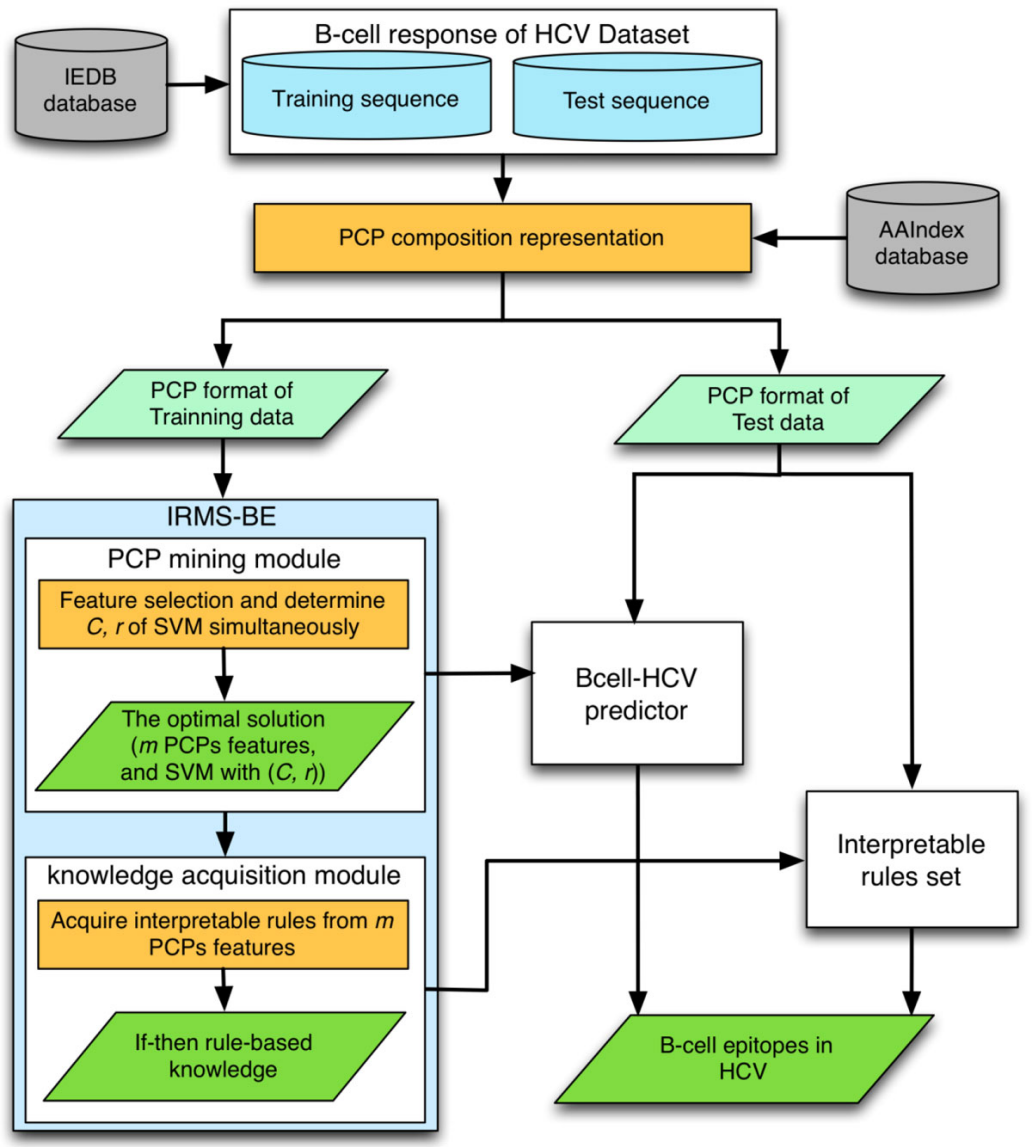
**The framework of the proposed IRMS-BE**. Seven main parts are in this block diagram, comprising: 1) B-cell response of HCV Dataset, 2) PCP composition representation, 3) IRMS-BE system, 4) PCP mining module, 5) Knowledge acquisition module, 6) Bcell-HCV predictor and 7) Interpretable rules set.

This work retrieved experimentally validated B-cell response of HCV dataset (BR-HCV) from the immune epitope database (IEDB) [22]. In order to provide insights into the mechanism of B-cell epitopes of HCV and improve the prediction accuracy, an interpretable rule mining system of B-cell epitopes (IRMS-BE) is proposed which consists of physiochemical property (PCP) mining module to select informative PCPs and knowledge acquisition module to extract several if-then rule-based knowledge of predicting B-cell epitopes in HCV (Figure 2). A web server Bcell-HCV for predicting linear B-cell epitopes in HCV was implemented using the 34 informative PCPs and yields a test accuracy of 70.7%, which is superior to that of other SVM-based methods (66.5%) for identifying B-cell epitopes of HCV and the two general-purpose methods (49.9%). This work uses a feature knockout procedure [23] to analyze the efficiency of the 34 PCP features in predicting antigenic epitopes in HCV. The three most important properties (AAindex IDs: GEIM800102, ISOY800107, and SNEP660101) present the same difference (5.36%) in a feature knockout procedure. Among the three essential properties, the property of principal component I (AAindex ID: SNEP660101) is related to aromatic structures. The alpha-helix structure (AAindex ID: GEIM800102) plays an significant role in connecting HCV and the host cell and in facilitating HCV entry into host cells.

Furthermore, knowledge acquisition module can obtain 12 interpretable rules from top-five PCPs that have a prediction accuracy of 73.4% and sensitivity of 75.6%.

Finally, a conserved promising vaccine candidate, PDREMVLYQE, is identified from the top-50 B-cell epitopes of HCV for inclusion in a vaccine against HCV. The promising candidate is simultaneously considering induce antibodies and neutralize as broad as possible.

The benefits of IRMS-BE lies in the incorporation of informative physicochemical properties and rule-based knowledge. Future research will be aimed at extending the IRMS-BE method to the prediction of B-cell epitopes in other viruses. Also, Bcell-HCV has been implemented as a web server and is available at http://e045.life.nctu.edu.tw/BcellHCV.

## Results

### Performance comparison with the sequence-based methods

**Figure 3:**
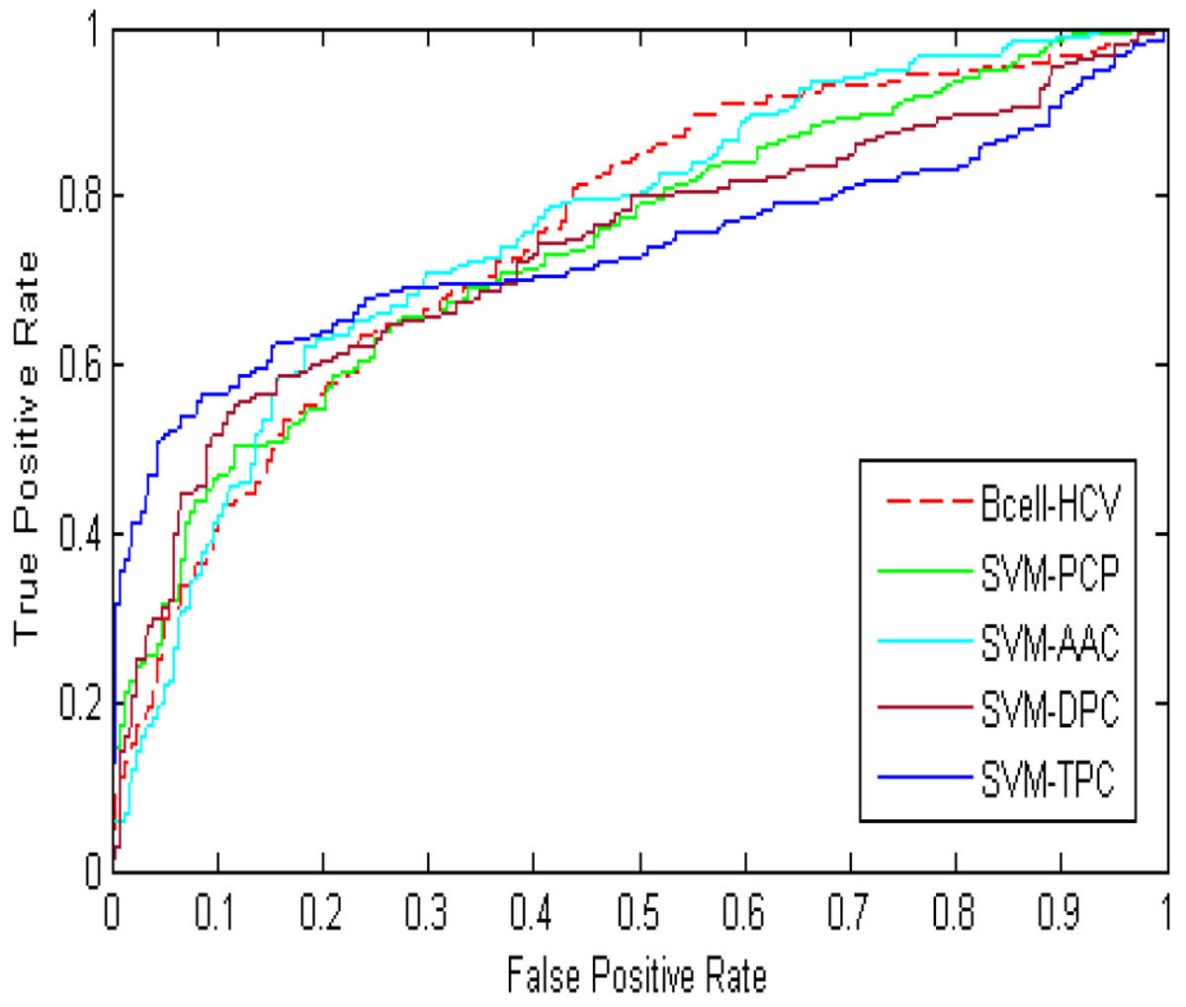
**The ROC curves of independent test on the BR-HCV^Te ^dataset**.

Using *m *= 34 physiochemical properties, the proposed prediction system Bcell-HCV produced training and independent test accuracies of 79.7% and 70.7%, where (*C*, γ) = (2^2^, 2^1^). Additionally, its training and test MCC values are 0.600 and 0.417, respectively. To evaluate the four SVM-based classifiers with 531 PCP features (referred to as SVM-PCP), 20 amino acid compositions (referred to as SVM-AAC), 400 dipeptide compositions (referred to as SVM-DPC), and 8000 tri-peptides (referred to as SVM-TPC), respectively, were evaluated in terms of prediction accuracy in 10-fold CV using the BR-HCV dataset. The best values for parameters γ and *C *in the SVM-based classifier were determined using a step-wise approach from γ ∈ {2^-15^, 2^-13^, ..., 2^16^} and *C*∈ {^-15^, 2^-13^, ..., 2^16^}. As shown in Table 2 the SVM-PCP classifier achieved accuracies of 74.80% and 65.50% when applied to the BR-HCV^Tr ^and BR-HCV^Te ^datasets, respectively, where (*C*, γ) = (2^1^, 2^-3^). Among the four SVM-based classifiers, SVM-PCP presented the second best performance after that of Bcell-HCV. These experimental results demonstrate that the prediction system Bcell-HCV outperforms the SVM-based methods (70.7% compared with 66.5% for test accuracy). Furthermore, two elegant and general-purpose methods [12,24] for predicting linear B-cell epitopes, ABCpred [12] and LBtope [24], using the BR-HCV^Te ^dataset were compared with Bcell-HCV. The results indicate that Bcell-HCV is better than the SVM-based methods for B-cell epitopes of HCV and the two general-purpose methods (Table 2). To prevent the threshold biased, the ROC curves are plotted using the BR-HCV^Te ^dataset (Figure 3).

**Table 2:** Prediction performance comparisons between Bcell-HCV and representive methods using the BR-HCV dataset.

Methods	Cross-validation on the BR-HCV^Tr ^dataset					Independent test on the BR-HCV^Te ^dataset				
	
	SP	SE	AUC	MCC	ACC	SP	SE	AUC	MCC	ACC
Bcell-HCV (this study)	0.864	0.731	0.97	0.596	79.6%	0.764	0.651	0.76	0.417	70.7%
SVM-PCP	0.903	0.862	0.95	0.766	87.9%	0.589	0.721	0.74	0.313	66.5%
SVM-AAC	0.870	0.797	0.99	0.662	83.1%	0.620	0.709	0.76	0.331	66.5%
SVM-DPC	0.934	0.889	0.98	0.811	90.5%	0.616	0.713	0.74	0.331	66.5%
SVM-TPC	0.948	0.895	0.96	0.831	91.5%	0.078	0.992	0.73	0.173	49.9%
LBtope (SVM)	-	-	-	-	-	0.162	0.841	-	-0.042	49.9%
ABCPred (ANN)	-	-	-	-	-	0.823	0.166	-	-0.015	49.4%

### Ranking the identified physiochemical properties

**Figure 4:**
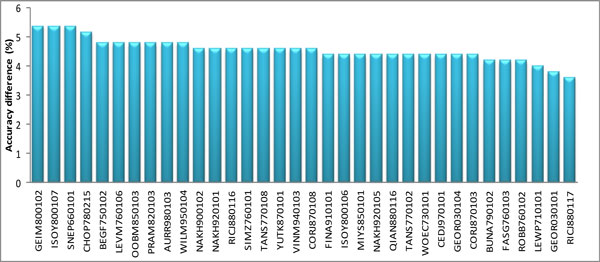
**The m = 34 PCP features ranked by the accuracy difference (Dj)**. The accuracy difference ***D_j _= Acc - Acc_j_***, for all ***j = 1, 2, ..., m***. The overall accuracy ***Acc ***is obtained using ***m ***PCP features, ρ = {ρ_1_, ρ_2_ , ... , ρ_m_ }. The feature-knockout accuracy ***Acc_j _***is obtained using the remaining ***m***-1 features after excluding the ***j***-th feature, where the ***m***-1 features from the ρ group.

The work adopted a feature-knockout approach [23] to rank the efficiency of *m *= 34 physiochemical properties (PCPs) in the prediction of linear B-cell epitopes. The most effective PCP has maximum accuracy difference (*D*_j _= *Acc - Acc*_j_, for all *j *= 1, 2,..., *m*) between overall accuracy and feature-knockout accuracy. Overall accuracy *Acc *is obtained using all *m *= 34 PCPs, ρ = {ρ_1_, ρ_2_,..., ρ_m_}. Feature-knockout accuracy *Acc*_j _is obtained by employing an SVM with *m-*1 PCPs in the ρ group. The group ρ contains *m*-1 PCPs except for ρ_j_. Figure 4 displays the *m *= 34 accuracy differences and rank in decreasing order. The top-10 PCPs are listed in Table 3 and relevant information associated with the 34 PCPs is listed in Supplementary Table 1. Top-three physiochemical properties are GEIM800102, ISOY800107, and SNEP660101, which achieve a maximum accuracy difference, is 5.36%.

**Table 3:** Definition of the top-10 properties ranked by the accuracy differences.

Rank	AAindex ID	Description	Difference
1	GEIM800102^S^	Alpha-helix indices for alpha-proteins (Geisow-Roberts, 1980)	5.360
2	ISOY800107^S^	Normalized relative frequency of double bend (Isogai et al., 1980)	5.360
3	SNEP660101	Principal component I (Sneath, 1966)	5.360
4	CHOP780215^S^	Frequency of the 4th residue in turn (Chou-Fasman, 1978b)	5.165
5	BEGF750102^S^	Conformational parameter of beta-structure (Beghin-Dirkx, 1975)	4.777
6	LEVM760106	van der Waals parameter R0 (Levitt, 1976)	4.777
7	OOBM850103	Optimized transfer energy parameter (Oobatake et al., 1985)	4.777
8	PRAM820103	Correlation coefficient in regression analysis (Prabhakaran-Ponnuswamy, 1982)	4.777
9	AURR980103^S^	Normalized positional residue frequency at helix termini N" (Aurora-Rose, 1998)	4.777
10	WILM950104	Hydrophobicity coefficient in RP-HPLC, C18 with 0.1%TFA/2-PrOH/MeCN/H2O (Wilce et al. 1995)	4.777

S: with secondary structure propensity;

The 34 properties contain 10 related to secondary structure, which are marked with "s", as shown in Additional File 1: Table S1. These include the conformational parameter of beta structure (AAindex ID BEGF750102), which is ranked fifth. The hydrophobicity coefficient in RP-HPLC, C8 with 0.1%TFA/MeCN/H2O (AAindex ID: WILM950102) is ranked tenth, which is in agreement with results obtained using AAP [15], suggesting that this property is efficient in the discrimination of linear B-cell epitopes and non B-cell epitopes.

### Rule-based knowledge

**Figure 5:**
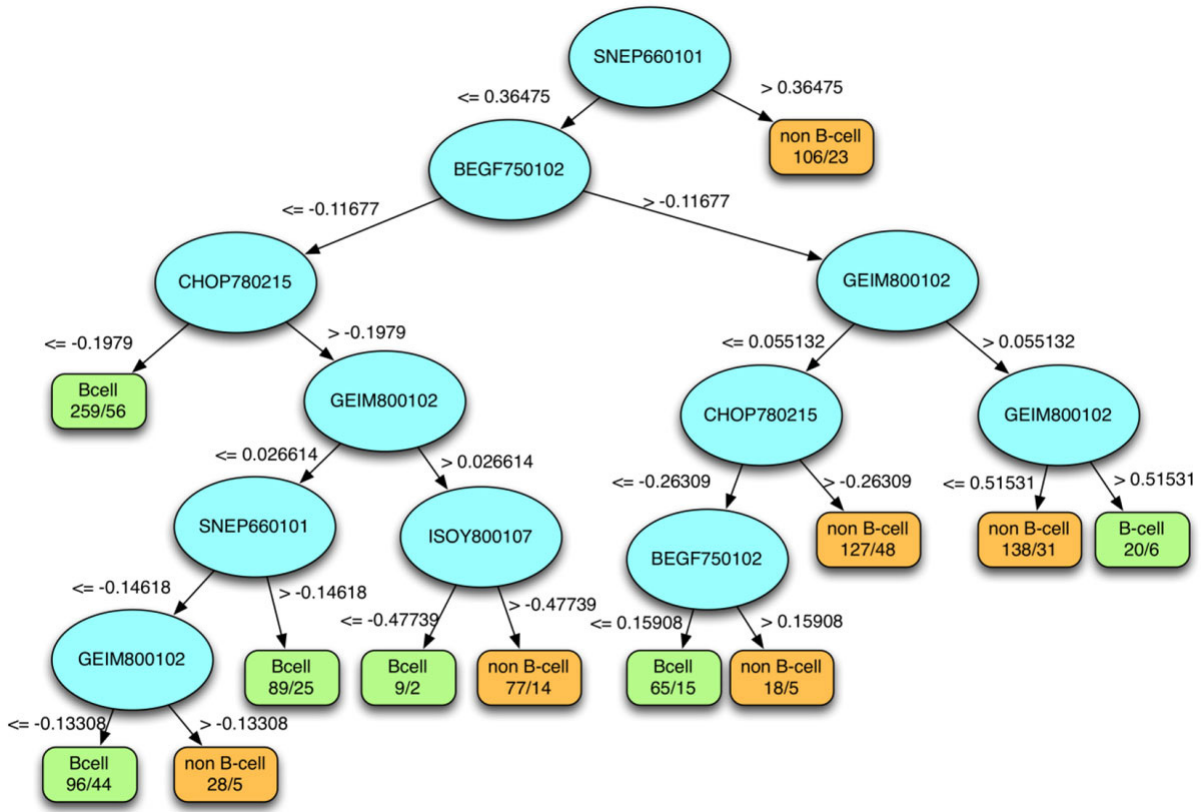
**A decision tree with top-5 features and pruning confidence level higher than 25%**. The accuracy of classifying the training dataset by using the top-5 features to construct decision tree is 73.4%.

This work presents a knowledge acquisition module based on the decision tree method C5.0, an improved version of C4.5 [25] to obtain insight into HCV antigenic epitopes. Knowledge can be obtained from two aspects: 1) the identification of informative physicochemical properties, and 2) if-then rules for distinguishing between B-cell and non B-cell epitopes. The top-five most influential features for predicting B-cell epitopes of HCV are utilized to acquire the rule set and a corresponding decision tree. Figure 5 shows a constructed decision tree with pruning confidence level higher than 25%. The accuracy of classifying the training dataset using the constructed decision tree is 73.4%. Furthermore, a set of 12 interpretable rules, six for identifying B-cell epitopes and six for identifying non B-cell epitopes, are transformed from this tree (Additional File 2: Table S2).

Table 4 presents the six interpretable rules for identifying B-cell epitopes of HCV with top-five important physicochemical properties, and each rule comprises the different number of the criteria. If a query sequence meets all of the criteria in one rule, then it is identified to B-cell epitopes of HCV. The first rule, which covers 259 samples and the accuracy is 78.3%, is constructed by three properties (SNEP660101, BEGF750102, and CHOP780215). This rule has three criteria: (1) Principal component I (property SNEP660101), which is related to aromatic structures, equal or less than 0.364754. This rule means a query sequence with a low ratio of aromatic structures composition. (2) The conformational parameter of beta structure (property BEGF750102) is equal or less than -0.11677, which means a query sequence with a high ratio of beta structures composition. (3) The normalized value of CHOP780215 is equal or less than -0.1979, which means a query sequence with a low frequency of fourth residue in turn composition.

**Table 4:** Six if-then rules for identifying B-cell epitope of HCV using C5.0 and top-five important physicochemical properties.

#	Rule	Covered samples	Misclassified sample	Accuracy
1	SNEP660101 <= 0.36475 AND BEGF750102 <= -0.11677 AND CHOP780215 <= -0.1979	259	56	78.3%
2	SNEP660101 <= 0.36475 AND BEGF750102 <= -0.11677 AND CHOP780215 > -0.1979 AND GEIM800102 > 0.026614 AND ISOY800107 <= -0.47739	9	2	77.8%
3	SNEP660101 <= 0.36475 AND -0.11677 < BEGF750102 <= 0.15908 AND GEIM800102 <= 0.055132 AND CHOP780215 <= -0.26309	65	15	76.9%
4	-0.14618<SNEP660101 <= 0.36475 AND BEGF750102 <= -0.11677 AND CHOP780215 > -0.1979 AND GEIM800102 <= 0.026614	89	25	71.9%
5	SNEP660101 <= 0.36475 AND BEGF750102 > -0.11677 AND GEIM800102 > 0.51531	20	6	70.0%
6	SNEP660101 <= -0.14618 AND BEGF750102 <= -0.11677 AND CHOP780215 > -0.1979 AND GEIM800102 <= -0.13308	96	44	54.2%

The second rule, which covers 9 samples and the accuracy is 77.8%, is constructed by five properties (SNEP660101, BEGF750102, CHOP780215, GEIM800102, and ISOY800107) that comprises the following five criteria: (1) The normalized value of SNEP660101is equal or less than 0.364754, which means a query sequence with a low ratio of aromatic structures composition. (2) The conformational parameter of beta structure (property BEGF750102) is equal or less than -0.11677, which means a query sequence with a high ratio of beta structures composition. (3) The normalized value of CHOP780215 is larger than -0.1979, which indicates a query sequence with a high frequency of 4^th ^residues in turn composition. (4) The normalized value of GEIM800102 is larger than 0.026614, which means a query sequence with an intermediate ratio of alpha-helix. (5) Normalized relative frequency of double bend (property ISOY800107) is equal or less than -0.47739, which means a query sequence with a low rate of the double bend.

The third rule, which covers 65 samples and the accuracy is 76.9%, is constructed by four properties (SNEP660101, BEGF750102, GEIM800102, and CHOP780215). This rule has four criteria: (1) The normalized value of SNEP660101is equal or less than 0.364754, which means a query sequence with a low ratio of aromatic structures composition. (2) The conformational parameter of beta structure (property BEGF750102) is larger than -0.11677 and less or equal 0.15908, which means a query sequence with a high ratio of beta structures composition. (3) The normalized value of GEIM800102 is less or equal than 0.055132, which means a query sequence with a low rate of alpha-helix. (4) The normalized value of CHOP780215 is less or equal than -0.26309, which means a query sequence with a low frequency of fourth residues in turn composition.

The fourth rule, which covers 89 samples and the accuracy is 76.9%, is constructed by four properties (SNEP660101, BEGF750102, CHOP780215, and GEIM800102). This rule has four criteria: (1) The normalized value of SNEP660101is larger than -0.14618 and equal or less than 0.364754, which means a query sequence with a low ratio of aromatic structures composition. (2) The conformational parameter of beta structure (property BEGF750102) is equal or less than -0.11677, which means a query sequence with a high ratio of beta structures composition. (3) The normalized value of CHOP780215 is larger than -0.1979, which indicates a query sequence with a high frequency of fourth residue in turn composition. (4) The normalized value of GEIM800102 is less or equal than 0.026614, which means a query sequence with a low ratio of the alpha-helix.

The fifth rule, which covers 20 samples and the accuracy is 70.0%, is constructed by three properties (SNEP660101, BEGF750102, and GEIM800102). This rule has three criteria: (1) Principal component I ((AAindex ID: SNEP660101), which is related to aromatic structures, equal or less than 0.364754, which means if a query sequence with a low ratio of aromatic structures composition, (2) The conformational parameter of beta structure (AAindex ID: BEGF750102) is larger than -0.11677, which means if a query sequence with a high ratio of beta structures composition (3) The normalized value of GEIM800102 is greater than 0.51531, which means a query sequence with a high rate of alpha-helix.

The sixth rule, which covers 96 samples and the accuracy is 54.2%, is constructed by four properties (SNEP660101, BEGF750102, CHOP780215, and GEIM800102). This rule has three criteria: (1) Principal component I (AAindex ID: SNEP660101), which is related to aromatic structures, equal or less than -0.14618, which means a query sequence with a low ratio of aromatic structures composition, (2) The conformational parameter of beta structure (AAindex ID: BEGF750102) is less and equal than -0.11677, which means a query sequence with a low ratio of beta structures composition (3) The normalized value of GEIM800102 is less than -0.13308, which means a query sequence with a low ratio of the alpha-helix formation.

### Identifying promising vaccine candidates

Sequence variability of neutralizing epitopes is considered to be a major obstacle to vaccine development [26]. Owing to the rapid change of antigenic profile of HCV, the promising vaccine candidate is identified from B-cell epitopes of HCV by the two-stage procedure: 1) making the neutralized range as broad as possible and 2) maximizing the ability to induce antibodies. To analyze the ranges of the top-*n *B-cell epitopes of HCV using a phylogenetic tree, the procedure is described below.

Step 1) Select the top-*n *B-cell epitopes in accordance with prediction scores of HCV using the prediction system Bcell-HCV.

**Figure 6:**
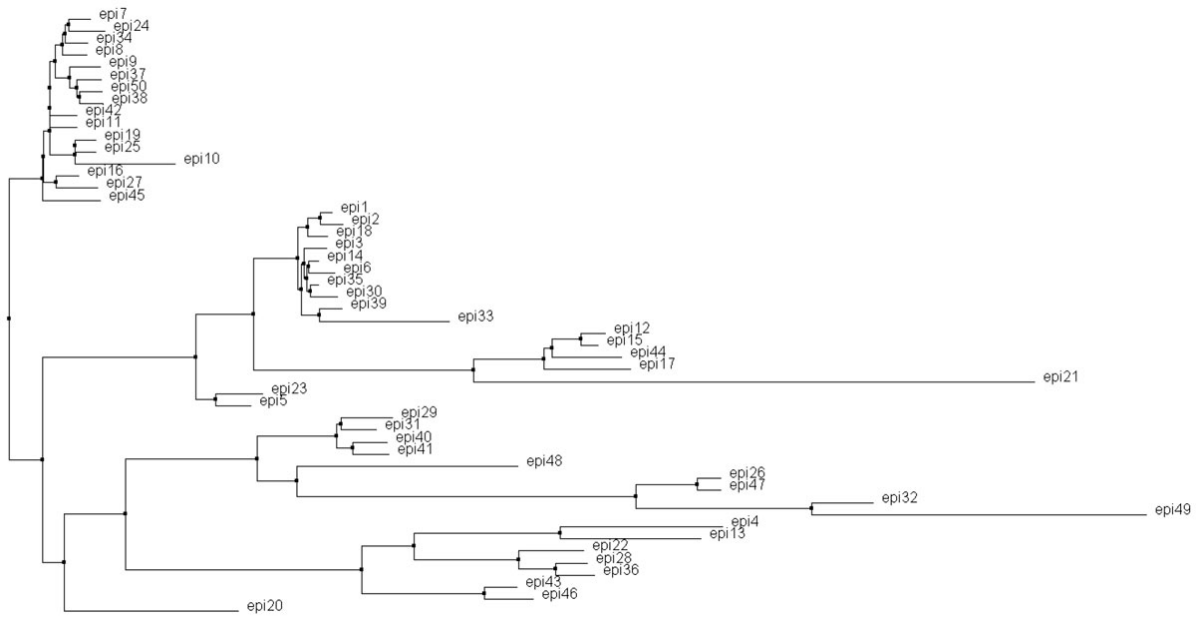
**A phylogenetic tree with top-n B-cell epitopes of HCV**. The phylogenetic tree is generated for analyzing the broadness of top-n B-cell epitopes of HCV using the neighbor joining tree with the BLOSUM62 scoring matrix from the Jalview tool. ***n ***= 50 in this example.

Step 2) Use these *n *B-cell epitopes of HCV to generate a phylogenetic tree (Figure 6) by applying the BLOSUM62 scoring matrix with the Jalview tool [27].

Step 3) Calculate the divergence is calculated by summing the distances of all edges in the corresponding phylogenetic tree.

Step 4) (Termination test) If *n *!= *k *then *n *= *n*+10 and go to the Step1. Otherwise, stop the algorithm. The value of *k *is determined by the specified threshold (*T*) for the average score of top-*n *linear B-cell epitopes. In this work, *T*= 0.95 is used.

In this work, the phylogenetic tree of the top-50 epitopes with the highest divergence (963.33) which denotes the neutralize range of the identified vaccine candidate is obtained (Additional File 3: Table S3). The detailed information of the top-50 B-cell epitopes of HCV is listed in Additional File 4: Table S4.

**Figure 7:**
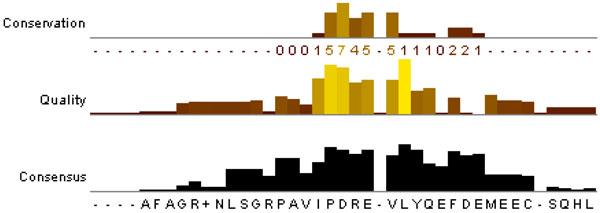
**The motif analysis using the MAFFT tool**. The MAFFT tool can analyze the conserved motif from the top-50 high-confidence B-cell epitopes of HCV. As a result, the conserved motif is PDRE-VLYQE.

The following procedure performs the second stage for maximizing the ability to induce antibodies:

Step 1) Use the MAFFT tool [28] to obtain the conserved motif (PDRE-VLYQE) in Figure 7 from the top-50 B-cell epitopes of HCV. The illustrated example is PDRE-VLYQE, shown in Figure 7.

Step 2) Insert every one of the 20 amino acids into the gap of the conserved motif (PDRE-VLYQE), to generate 20 peptides.

Step 3) The best one of the 20 peptides is a vaccine candidate (PDREMVLYQE) in accordance with prediction scores using the Bcell-HCV (Table 5).

**Table 5:** Prediction scores of 20 vaccine candidates from the conserved motif.

No	Sequence	Score
1	PDRE**M**VLYQE	0.92
2	PDRE**I**VLYQE	0.91
3	PDRE**F**VLYQE	0.88
4	PDRE**V**VLYQE	0.87
5	PDRE**N**VLYQE	0.83
6	PDRE**C**VLYQE	0.80
7	PDRE**A**VLYQE	0.79
8	PDRE**S**VLYQE	0.77
9	PDRE**L**VLYQE	0.73
10	PDRE**H**VLYQE	0.72
11	PDRE**T**VLYQE	0.68
12	PDRE**G**VLYQE	0.67
13	PDRE**K**VLYQE	0.66
14	PDRE**Y**VLYQE	0.63
15	PDRE**Q**VLYQE	0.60
16	PDRE**W**VLYQE	0.60
17	PDRE**E**VLYQE	0.56
18	PDRE**R**VLYQE	0.51
19	PDRE**D**VLYQE	0.50
20	PDRE**P**VLYQE	0.48

## Discussion

The one of the major challenges of designing a peptide-based vaccine system is related to the collection of non-epitopes. Some residues in epitope are incorrectly annotated as non-epitopes led to overestimation problem that is overestimating of false positive rate. Most researchers [21,29,30] dealt with this overestimation problem by extracting a random sample of the protein sequences in Swiss-Prot [31]. This work solves this overestimation problem using experimentally verified non B-cell epitopes derived from IEDB [22]. The sequence pattern of linear B-cell epitopes of similar pathogens are quite different leading to the underperformance of general-purpose computational methods [20,21] for predicting B-cell epitopes in HCV, which is consistent with the independent test results of two general-purpose tools [12,24]. Hence, developing a virus-specific tool is important to accurately identify linear B-cell epitopes in a particular virus. To our knowledge, this is the first HCV-customized report showing that predicting antigenic epitopes in HCV, analysis of informative physicochemical properties, and identifying promising vaccine candidate from two views which are inducing antibodies and neutralizing as broad as possible.

Analyzing the relationships among the 34 identified physicochemical properties and the estimated epitope sequences provided some insight into linear B-cell epitopes in HCV. As shown in Table 3 the three most influential properties (obtained using AAindex IDs GEIM800102, ISOY800107, and SNEP660101) have the same difference in accuracy (5.36%), which clearly indicates their significant contribution to the prediction of HCV antigenic epitopes. These include alpha and turn propensities, hydrophobicity, and aromatic properties.

The E2 segment plays a significant role in HCV connection and entry into host cells, and the alpha-helix structure (GEIM800102) of epitopes has a strong influence on this connection. For example, one of the central binding regions in the E2 protein is formed by hydrophobic interactions on the alpha-helix, which is located at the C-terminal [32]. Furthermore, the spatial arrangement of the components at E2 of HCV is found to deviate significantly from the corresponding complexes with neutralizing antibodies [33].

Antibodies that target the two hypervariable regions of the E2 segment include HVR1 and HVR2 [34]. These two parts on the E2 segment contain conservative residues, such as Thr^2, ^Gly^6, ^Gly^23^, and Gln^26^, which are polar amino acids that form hydrogen bonds [35]. Furthermore, in a work by Kong et al that analyzed antibodies of HCV, the major antibody binding sites were found to be conserved, and the residues that were substituted in those sites showed similar hydrophobicity [36]. For HCV, the interaction between the antibody and epitope depends on specific residues from the hydrophobic face of the epitope. Accordingly, replacing these with polar or charged residues could weaken or eliminate the interaction between the antibody and the antigen [36].

**Figure 8:**
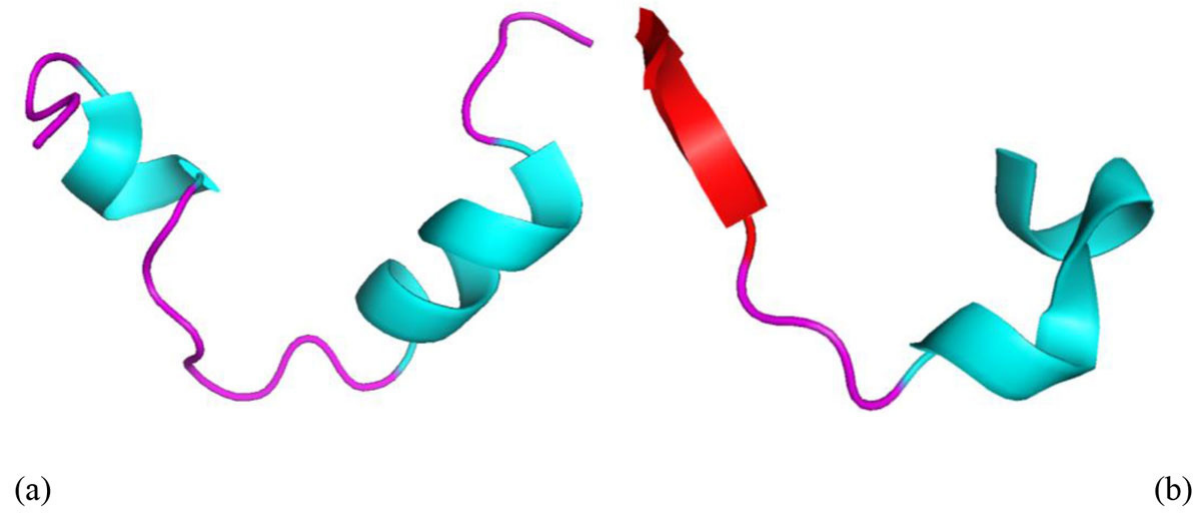
**The 3D structures of the linear B-cell epitope and non B-cell epitope**. The 3D structures can distinguish when considering the top propensity of Alpha-helix indices for alpha-proteins with AAindex ID: GEIM800102 (a) The structures of linear B-cell epitope are mainly composed of helixes (blue). (b) But the structures of non B-cell epitopes contain beta-sheets (red, at the left) also to helixes (blue, at the right).

To investigate the property of the alpha-helix structure (GEIM800102), the ps2 protein structure prediction server (http://ps2.life.nctu.edu.tw/) to predict the 3D structure of the query epitopes. The PyMOL (http://sourceforge.net/projects/pymol/) molecular visualization system was then used to present molecular visualization in 3D. For example, in Figure 8(a), "YPGHVSGHRMAWDMM" is a linear B-cell epitope which mainly forms a helix structure (blue), whereas "RLWHYPCTINYTIFKI" is a non B-cell epitope that possesses an alpha structure (red) as well as a helix structure (blue).

The property of principal component I (AAindex ID: SNEP660101) is related to aromatic structures, which were discovered to play a fundamental role in the interaction between antibodies and epitopes. The side chain of two aromatic residues, Phe^442^, and Tyr^443^, are exposed on the same side of the helix in the E2 protein [37] and close to the binding residues, Leu^438^, Ala^439^, and Leu^441^. Systematic studies on mutagenesis have shown that only Leu^438 ^and Ala^439 ^have the ability to tolerate mutations. This means that the mutation of those two residues should not damage the ability of the virus to bind to host cells, whereas a mutation in one of the two aromatic residues would render the HCV virus non-functional [8,37].

In conclusion, the binding sites of HCV antibodies are located in a predominantly hydrophobic cavity with aromatic residues that play a critical role in the interaction with antibodies [36]. Some residues can adjust the shape space of epitopes in the connection between antibodies and epitopes. For instance, the small amino acids glycine and proline are found interspersed between aromatic residues, which can alter the geometry of the hydrophobic region to fit with various antibodies [38].

## Conclusions

Development of an effective vaccine against hepatitis C virus (HCV) is a complex task owing to the variability of this RNA virus. Recently, development of HCV vaccines has mainly focused on T-cell immune response. However, B-cell epitopes that can stimulate B-cell response is one of the major tasks of peptide-based vaccine development. This work proposes an interpretable rule mining system IRMS-BE for extracting interpretable rules using informative physicochemical properties and a web server Bcell-HCV for predicting linear B-cell epitopes of HCV. Finally, a conserved promising vaccine candidate, PDREMVLYQE, is identified for inclusion in a vaccine against HCV.

## Methods

The block diagram in Figure 1 outlines the steps involved in modeling with the proposed IRMS-BE system, including 1) Datasets, 2) representing PCP composition, 3) IRMS-BE system, 4) PCP mining module, 5) knowledge acquisition module, 6) predicting B-cell epitopes of HCV and 7) interpretable rules set. These steps were applied in this work, and detailed descriptions are in the following sub-sections.

### Datasets

A B-cell response of HCV (referred to as BR-HCV) dataset was established to evaluate IRMS-BE and Bcell-HCV. BR-HCV dataset were collected from the immune epitope database (IEDB) [22] (version 2.12 released on Dec. 16, 2013), which contains data related to antibodies and T-cell epitopes in humans, non-human primates, rodents, and other animal species. The latest version (version 2.3) was released on July 2, 2014. The BR-HCV dataset was created as follows.

Step 1) The source organism "Hepatitis C virus" was used to find the B-cell response of HCV sequences. This result involved the collection of 8009 B-cell response sequences, including 4041 linear B-cell epitopes (positives) and 3968 non B-cell epitopes (negatives), as shown in Additional File 5: Figure S1.

Step 2) Epitopes with 10- to 20-mers lengths were selected. The epitopes annotated with a greater number of 'positive' (i.e. as opposed to 'negative') results were regarded as positive samples. Conversely, peptide sequences with more 'negative' results were considered as negative. For example, Additional File 6: Figure S2 (a) and (b) show the epitope 'YLLPRRGPRL' was considered a positive sample because most of the experiment results were positive. The epitope 'DLMGYIPLV', as shown in Additional File 6: Figure S2 (c) was considered a negative sample is showing the opposite criteria.

Step 3) Redundant samples (i.e., epitopes that shared 20% or more sequence identity with any other peptides in the same subset) were removed from the benchmark data sets to create a non-redundant dataset. This work used the PICES [39] culling program, which resulted in the collection of 1548 B-cell response sequences, 774 positives and 774 negatives, to establish the BR-HCV dataset

Step 4) The BR-HCV dataset was divided into two parts (BR-HCV^Tr ^and BR-HCV^Te^) for training and independent test. The BR-HCV^Tr ^and BR-HCV^Te ^datasets, which were kept at a ratio of 2:1, contained 1032 (516 positives and 516 negatives) and 516 (258 positives and 258 negatives) B-cell response sequences, respectively.

### Representation of PCP composition

Physicochemical properties (PCPs), also referred to as propensity, are the most intuitive feature associated with biochemical reactions and are widely used in the field of bioinformatics. This work represents each peptide sequence for an *l*-dimensional profile, where the value of each amino acid is obtained from the AAindex [40] database to encode a particular PCP feature. The *l*-dimensional profiles are transformed into the *N_AAindex_*-dimensional feature vectors (referred to as PCP composition), where *N_AAindex _*= 531 physicochemical properties can be obtained from http://www.genome.ad.jp/aaindex[40]. In this work, *l *= 10, 11 to 20 are used. Finally, all values of the feature vectors are normalized into [-1, 1] before being input into the SVM.

### IRMS-BE system

An interpretable rule mining system of B-cell epitopes (IRMS-BE) including physiochemical properties (PCPs) mining module and knowledge acquisition module is proposed. PCPs mining module selects 34 informative PCPs from 531 physiochemical properties and determines the values of *C *and *γ *of the used SVM simultaneously based on an inheritable bi-objective genetic algorithm [41]. The knowledge acquisition module is based on the 34 informative PCPs, a decision tree method C5.0 [42] was used to extract if-then rule-based knowledge for the biologist to understand the mechanism of B-cell epitopes in HCV.

### PCP mining module

To identify minimal number (*m*) out of 531 PCP features while establishing an SVM-based training classifier (referred to as PCP mining) with maximal accuracy is a bi-objective combinatorial optimization problem [43]. Physicochemical property mining module (PCP mining module) solved this optimization problem by utilizing an inheritable bi-objective combinatorial optimization genetic algorithm (IBCGA). The PCP mining module to consider internal correlations among relevant features rather than focusing on individual features [44].

To select a minimal set of *m *informative PCPs from *n *= 531 PCPs while maximizing the prediction accuracy of using these *m *features for designing an SVM classifier is a bi-objective combinatorial optimization problem C(*n, m*). To cope with this large parameter optimization problem, the inheritable bi-objective combinatorial genetic algorithm (IBCGA) is used [9]. The IBCGA can simultaneously obtain a set of solutions, *S_r_*, where *r *= *r_start_, r_start_*+1, ..., *r_end _*in a single run using an inheritance mechanism to efficiently search for a solution *S_r+1 _*to C(*n, r*+1) by inheriting a good solution *S_r _*to C(*n, r*). On the other hand, the IBCGA using an intelligent evolutionary algorithm (IEA) [10] which can efficiently solve large parameter optimization problems is good at deriving an optimized SVM model with the feature selection. The high performance of IEA mainly arises from using an orthogonal array based crossover operation with a systematic reasoning ability instead of the traditional crossover operation with a generate-and-go mechanism. The detailed method can refer to the work [9]. Considering the purposes of this work, involving both model selection and estimation of prediction errors, the cross-validation scheme is used with an SVM-based classifier of this PCP mining module. To reduce computational costs, PCP mining module used the prediction accuracy (ACC) of 10-fold CV to serve as the fitness function of IBCGA [24] for the entire training set. The evaluation of binary predictions involves the use of several quality measures: accuracy (ACC), sensitivity (SE), specificity (SP), and the Matthews correlation coefficient (MCC):

(1) $$\begin{array}{c} \text{ACC}=\left(\text{TP}+\text{TN}\right)/\left(\text{TP}+\text{FP}+\text{TN}+\text{FN}\right)\\ \text{SE}=\text{TP}/\left(\text{TP}+\text{FN}\right)\\ \text{SP}=\text{TN}/\left(\text{TN}+\text{FP}\right)\\ \text{MCC}=\left(\text{TP}\times \text{TN}-\text{FP}\times \text{FN}\right)/\sqrt{\left(\text{TP}+\text{FP}\right)\times \left(\text{TP}+\text{FN}\right)\times \left(\text{TN}+\text{FP}\right)\times \left(\text{TN}+\text{FN}\right)} \end{array}$$

where TP and TN are the numbers of correctly predicted linear B-cell epitopes and non B-cell epitopes, respectively. FP and FN are the numbers of incorrectly predicted linear B-cell epitopes and non B-cell epitopes, respectively. MCC is often used to evaluate the balance of model prediction.

The input of the PCP mining module is a training set of protein sequences belonging to two classes: positives and negatives. The output contains a set of *m *selected PCP features and an SVM-based classifier with associated parameter settings, *γ*and *C*. Protocols for the PCP mining module are as follows:

Step 1) Each sample is represented as an *n*-dimensional feature vector *P *= [*p_1_, p_2_, ..., p_n_*] using the composition of PCPs

Step 2) The IBCGA-chromosome consists of binary genes *f_i _*from which to select PCP features and two 4-bit genes for encoding kernel parameter (γ) and cost parameter (*C*). The corresponding feature *p_i _*(the *i*-th PCP feature) is excluded from the SVM classifier if *f_i _*= 0, and is included if *f_i _*= 1. Let *m *be the sum of *f_i_*. The γ > 0 determines how the samples are transformed into a high-dimensional search space. The cost parameter C>0 of the SVM classifier adjusts the penalty of total error. These two parameters C and γ must be tuned to get the best prediction performance. In this work, γ ∈ {2^-15^, 2^-13^, ..., 2^16^}and *C*∈ {2^-15^, 2^-13^, ..., 2^16^}.

Step 3) The fitness function is the prediction accuracy of 10-fold CV using the LIBSVM classifier [47] with *m *selected PCP features, γ and *C *by decoding the IBCGA-chromosome. In this work, a popular kernel function that is radial basis function exp (− γ ||*x^i ^x^j^*||^2^) is adopted. The *x^i ^*and *x^j ^*are training samples and γ is a kernel parameter. The parameter settings of IBCGA are given in Additional File 7: Table S5.

Step 4) All solutions for *S_r _*from *r*=*r_start _*to *r*_end _are obtained using IBCGA. Let *S_m _*be the most accurate solution with *m *selected PCP features among all solutions from *C*(*n, r_start_*) to *C*(*n, r_end_*) search space. In this work, *r_start _*= 10 and *r*_end _= 40 are used.

Step 5) IBCGA use mechanisms of randomization and are therefore characterized as non-deterministic based on the fact that results of individual runs are not the same always. Therefore, Steps 3) and Step 4) are performed for *R *independent runs to obtain the best *R *number of discrete runs to get the best *R *solutions, In this work, *R *= 30 is used.

### The knowledge acquisition module

Decision tree algorithms are valuable to obtain the rule-based knowledge since the tree can generate if-then rules. In this work, the method C5.0 is employed to construct decision tree classifier and acquire interpretable rule set for analyzing hepatitis C virus. The decision tree is constructed using ranked properties, which selected by information gain, and can be used to select properties, according to the ranks of properties. Nevertheless, the selected properties have no interaction between each property and the influence of properties should be considered individually. For acquiring the general and interpretable rules, the pruning process is applied to avoid the over-fitting problem and the threshold value of confidence is set to 25%. The final tree can transfer to if-then rules that one rule is corresponded to one leaf node. The covered samples of a rule are the samples in one leaf node.

### Prediction system of B-cell epitopes of HCV

To provide prediction service to the scientific community, we have developed a user-friendly web server Bcell-HCV based on 34 informative physicochemical properties and optimized parameters (*C, γ*) of SVM classifier in this study. The 34 informative physicochemical properties were selected by the PCP mining training module.

## Availability

The web server Bcell-HCV can deal with the amino acid sequences of HCV antigens in FASTA format. Users can input the size of sliding window and the threshold of prediction score for screening high-confidence putative epitopes. The output is the identified B-cell epitopes with location information and their prediction scores. The high score implies a great probability for the peptide to be a B-cell epitope of HCV. The web server is freely available at http://e045.life.nctu.edu.tw/BcellHCV/

## Competing interests

The authors declare that they have no competing interests.

## Authors' contributions

Wen-Lin Huang (WLH) and Ming-Ju Tsai (MJT) designed the system, participated in manuscript preparation, and carried out the detail work. MJT, Kai-Ti Hsu, Jyun-Rong Wang (JRW) and Yi-Hsiung Chen (YHC) designed the system and implemented programs. Also, Shinn-Ying Ho (SYH) and WLH supervised the whole project and participated in manuscript preparation. All authors have read and approved the final manuscript.

## Supplementary Material

Additional file 1 **Table S1**. Definition of the 34 properties ranked by the accuracy differences. Click here for file

Additional file 2 **Table S2**. Rule-based knowledge of Bcell-HCV prediction. Click here for file

Additional file 3 **Table S3**. Statistics of the top-k epitopes for the threshold > 0.95. Click here for file

Additional file 4 **Table S4**. The top-50 B-cell epitopes of HCV for analyzing the conserved motif and constructing a phylogenetic tree are listed. The order of peptide IDs are sorted using the prediction score. Click here for file

Additional file 5 **Figure S1**. Figure S1 (a) HCV source organism. (b) A total of 8009 non-redundant linear B-cell epitopes and non B-cell epitopes are obtained. Click here for file

Additional file 6 **Figure S2**. Figure S2 Collect relevant antigenic sequences with (b) B-cell assay data, based on the (b) positive samples and (c) negative samples. Click here for file

Additional file 7 **Table S5**. The control parameters of IBCGA used. Click here for file

## Declaration

Publication charges for this article have been funded by the National Science Council of Taiwan under the contract number MOST-103-2221-E-009-117-

This article has been published as part of *BMC Medical Genomics *Volume 8 Supplement 4, 2015: Joint 26th Genome Informatics Workshop and 14th International Conference on Bioinformatics: Medical genomics. The full contents of the supplement are available online at http://www.biomedcentral.com/bmcmedgenomics/supplements/8/S4.
