# Supplementary material for: Prediction of linear B-cell epitopes of hepatitis C virus for vaccine development
Source: BMC Med Genomics. 2015 Dec 9;8(Suppl 4):S3. doi: 10.1186/1755-8794-8-S4-S3 (PMC4682406; doi:10.1186/1755-8794-8-S4-S3)
Supplement: Additional file 1 — Table S1. Definition of the 34 properties ranked by the accuracy differences. [file 1755-8794-8-S4-S3-S1.pdf]

Table S1

Definition of the 34 properties ranked by the accuracy differences

Rank	AAindex ID	Description	Difference
1	GEIM800102[S]	Alpha-helix indices for alpha-proteins (Geisow-Roberts, 1980)	5.360
2	ISOY800107[S]	Normalized relative frequency of double bend (Isogai et al., 1980)	5.360
3	SNEP660101	Principal component I (Sneath, 1966)	5.360
4	CHOP780215[S]	Frequency of the 4th residue in turn (Chou-Fasman, 1978b)	5.165
5	BEGF750102[S]	Conformational parameter of beta-structure (Beghin-Dirkx, 1975)	4.777
6	LEVM760106	van der Waals parameter R0 (Levitt, 1976)	4.777
7	OOBM850103	Optimized transfer energy parameter (Oobatake et al., 1985)	4.777
8	PRAM820103	Correlation coefficient in regression analysis (Prabhakaran-Ponnuswamy, 1982)	4.777
9	AURR980103[S]	Normalized positional residue frequency at helix termini N" (Aurora-Rose, 1998)	4.777
10	WILM950104	Hydrophobicity coefficient in RP-HPLC, C18 with 0.1%TFA/2-PrOH/MeCN/H2O (Wilce et al. 1995)	4.777
11	NAKH900102	SD of AA composition of total proteins (Nakashima et al., 1990)	4.583
12	NAKH920101	AA composition of CYT of single-spanning proteins (Nakashima-Nishikawa, 1992)	4.583
13	RICJ880116	Relative preference value at C' (Richardson-Richardson, 1988)	4.583
14	SIMZ760101	Transfer free energy (Simon, 1976)	4.583
15	TANS770108	Normalized frequency of zeta R (Tanaka-Scheraga, 1977)	4.583
16	YUTK870101	Unfolding Gibbs energy in water, pH7.0 (Yutani et al., 1987)	4.583
17	VINM940103	Normalized flexibility parameters (B-values) for each residue surrounded by one rigid neighbours (Vihinen et al., 1994)	4.583
18	CORJ870108	TOTLS index (Cornette et al., 1987)	4.583
19	FINA910101[S]	Helix initiation parameter at posision i-1 (Finkelstein et al., 1991)	4.389
20	ISOY800106[S]	Normalized relative frequency of helix end (Isogai et al., 1980)	4.389
21	MIYS850101	Effective partition energy (Miyazawa-Jernigan, 1985)	4.389
22	NAKH920105	AA composition of MEM of single-spanning proteins (Nakashima-Nishikawa, 1992)	4.389
23	QIAN880116[S]	Weights for beta-sheet at the window position of -4 (Qian-Sejnowski, 1988)	4.389
24	TANS770102 [S]	Normalized frequency of isolated helix (Tanaka-Scheraga, 1977)	4.389
25	WOEC730101	Polar requirement (Woese, 1973)	4.389
26	CEDJ970101	Composition of amino acids in extracellular proteins (percent) (Cedano et al., 1997)	4.389
27	GEOR030104	Linker propensity from 3-linker dataset (George-Heringa, 2003)	4.389
28	CORJ870103	PRIFT index (Cornette et al., 1987)	4.389
29	BUNA790102	alpha-CH chemical shifts (Bundi-Wuthrich, 1979)	4.194
30	FASG760103	Optical rotation (Fasman, 1976)	4.194
31	ROBB760102[S]	Information measure for N-terminal helix (Robson-Suzuki, 1976)	4.194
32	LEWP710101[S]	Frequency of occurrence in beta-bends (Lewis et al., 1971)	4.000
33	GEOR030101	Linker propensity from all dataset (George-Heringa, 2003)	3.806
34	RICJ880117	Relative preference value at C" (Richardson-Richardson, 1988)	3.612
