# Supplementary material for: Prediction of linear B-cell epitopes of hepatitis C virus for vaccine development
Source: BMC Med Genomics. 2015 Dec 9;8(Suppl 4):S3. doi: 10.1186/1755-8794-8-S4-S3 (PMC4682406; doi:10.1186/1755-8794-8-S4-S3)
Supplement: Additional file 2 — Table S2. Rule-based knowledge of Bcell-HCV prediction. [file 1755-8794-8-S4-S3-S2.pdf]

Table S2

Rule-based knowledge of Bcell-HCV prediction

#	Rule	B-Cell epitopes	Covered samples	Misclassified sample	Accuracy
1	SNEP660101 <= 0.36475 AND BEGF750102 <= -0.11677 AND CHOP780215 <= -0.1979	Y	259	56	78.3%
2	SNEP660101 <= 0.36475 AND BEGF750102 <= -0.11677 AND CHOP780215 > -0.1979 AND GEIM800102 > 0.026614 AND ISOY800107 <= -0.47739	Y	9	2	77.8%
2	SNEP660101 <= 0.36475 AND BEGF750102 > -0.11677 AND GEIM800102 <= 0.055132 AND CHOP780215 <= -0.26309 AND BEGF750102 <= 0.15908	Y	65	15	76.9%
4	SNEP660101 <= 0.36475 AND BEGF750102 <= -0.11677 AND CHOP780215 > -0.1979 AND GEIM800102 <= 0.026614 AND SNEP660101 > -0.14618	Y	89	25	71.9%
5	SNEP660101 <= 0.36475 AND BEGF750102 > -0.11677 AND GEIM800102 > 0.055132 AND GEIM800102 > 0.51531	Y	20	6	70.0%
6	SNEP660101 <= 0.36475 AND BEGF750102 <= -0.11677 AND CHOP780215 > -0.1979 AND GEIM800102 <= 0.026614 AND SNEP660101 <= -0.14618 AND GEIM800102 <= -0.13308	Y	96	44	54.2%
7	SNEP660101 > 0.36475	N	106	23	78.3%
8	SNEP660101 <= 0.36475 AND BEGF750102 <= -0.11677 AND CHOP780215 > -0.1979 AND GEIM800102 <= 0.026614 AND SNEP660101 <= -0.14618 AND GEIM800102 > -0.13308	N	28	5	82.1%
9	SNEP660101 <= 0.36475 AND BEGF750102 <= -0.11677 AND CHOP780215 > -0.1979 AND GEIM800102 > 0.026614 AND ISOY800107 > -0.47739	N	77	14	81.8%
10	SNEP660101 <= 0.36475 AND BEGF750102 > -0.11677 AND GEIM800102 <= 0.055132 AND CHOP780215 <= -0.26309 AND BEGF750102 > 0.15908	N	18	5	72.2%
11	SNEP660101 <= 0.36475 AND BEGF750102 > -0.11677 AND GEIM800102 <= 0.055132 AND CHOP780215 > -0.26309	N	127	48	62.2%
12	SNEP660101 <= 0.36475 AND BEGF750102 > -0.11677 AND GEIM800102 > 0.055132 AND GEIM800102 <= 0.51531	N	138	31	77.5%
