# Supplementary material for: Prediction of linear B-cell epitopes of hepatitis C virus for vaccine development
Source: BMC Med Genomics. 2015 Dec 9;8(Suppl 4):S3. doi: 10.1186/1755-8794-8-S4-S3 (PMC4682406; doi:10.1186/1755-8794-8-S4-S3)
Supplement: Additional file 3 — Table S3. Statistics of the top-k epitopes for the threshold > 0.95. [file 1755-8794-8-S4-S3-S3.pdf]

**Table S3**

Statistics of the top-k epitopes for the threshold > 0.95

| Top-k epitopes | Average scores | Divergence |
|---|---|---|
| 30 | 0.97 | 677.98 |
| 40 | 0.97 | 767.63 |
| 50 | 0.96 | 963.33 |
