# Supplementary material for: Prediction of linear B-cell epitopes of hepatitis C virus for vaccine development
Source: BMC Med Genomics. 2015 Dec 9;8(Suppl 4):S3. doi: 10.1186/1755-8794-8-S4-S3 (PMC4682406; doi:10.1186/1755-8794-8-S4-S3)
Supplement: Additional file 4 — Table S4. The top-50 B-cell epitopes of HCV for analyzing the conserved motif and constructing a phylogenetic tree are listed. The order of peptide IDs are sorted using the prediction score. [file 1755-8794-8-S4-S3-S4.pdf]

Table S4

The top-50 B-cell epitopes of HCV for analyzing the conserved motif and constructing a phylogenetic tree are listed. The order of peptide IDs are sorted using the prediction score.

ID	Sequence	ID	Sequence
epi1	PATVPDREVLYQEFDEMEEC	epi26	SRGNHVSPTHYVPESDAAAR
epi2	PATIPDREVLYREFDEMEEC	epi27	LTGKPAVVPDREILYQQFDE
epi3	PAVIPDREVLYQQFDEMEEC	epi28	ASHLPYIEQGMQLAEQFKQK
epi4	MEECASHLPYIEQGVQLAEQ	epi29	GRIILSGRPAIVPDRELLYQ
epi5	RPAVIPDREVLYQEFDEMEE	epi30	PAIIPDREALYQEFDEMEEC
epi6	PAIVPDREVLYQDFDEMEEC	epi31	GRIILSGRPAVIPDREVLYQ
epi7	LSGRPAIVPDREVLYQEFDE	epi32	AFASRGNHVSPTHYVPESDA
epi8	LSGRPAVIPDREVLYQEFDE	epi33	TVVAPDKEVLYEAFDEMEEC
epi9	LSGKPAVIPDREVLYREFDE	epi34	LSGRPAIIPDREVLYQEFDE
epi10	LSGKPATIPDREAVLYQEFD	epi35	PAIIPDREVLYQEFDEMEEC
epi11	LSGKPAVVPDREVLYQEFDE	epi36	ASHLPYIEQGMHLAEQFKQK
epi12	PDREVLYQQFDEMEECSKHL	epi37	LSGRPAVIPDREVLYREFDE
epi13	MEECSQAAPYIEQAQAIAHQ	epi38	LSGRPAIVPDREVLYREFDE
epi14	PAVVPDREVLYQEFDEMEEC	epi39	PAVIPDREVLYQAFDEMEEC
epi15	PDREVLYQEFDEMEECSQHL	epi40	GRIILSGRPAVIPDREVLYR
epi16	LTGKPAVIPDREVLYQEFDE	epi41	GRIVLSGRPAIIPDREVLYR
epi17	PDKEVLYQQYDEMEECSQAA	epi42	LSGKPAIIPDREVLYQEFDE
epi18	PAVIPDREVLYREFDEMEEC	epi43	SQAAPYIEQAQVIAHQFKEK
epi19	LSGKPATVPDREVLYQEFDE	epi44	PDREALYQQFDEMEECSASL
epi20	INQRTVVAPDKEVLYEAFDE	epi45	LSGKPAIIPDREALYQQFDE
epi21	QGMQLAEQLKQKALGLLQTA	epi46	SQAAPYIEQAQAIAHQFKEK
epi22	CASHLPYIEQGMQLAEQFKQ	epi47	SRGNHVSPAHYVPESDAAAR
epi23	KPAIIPDREVLYREFDEMEE	epi48	GRLHVNERAVVAPDKEVLYE
epi24	LSGRPAIVPDRELLYQEFDE	epi49	NRLIAFASRGNHVAPTHYVT
epi25	LSGKPATIPDREVLYQEFDE	epi50	LSGRPAVVPDREVLYREFDE
