# Supplementary material for: Prediction of linear B-cell epitopes of hepatitis C virus for vaccine development
Source: BMC Med Genomics. 2015 Dec 9;8(Suppl 4):S3. doi: 10.1186/1755-8794-8-S4-S3 (PMC4682406; doi:10.1186/1755-8794-8-S4-S3)
Supplement: Additional file 5 — Figure S1. Figure S1 (a) HCV source organism. (b) A total of 8009 non-redundant linear B-cell epitopes and non B-cell epitopes are obtained. [file 1755-8794-8-S4-S3-S5.pdf]

Figure S1

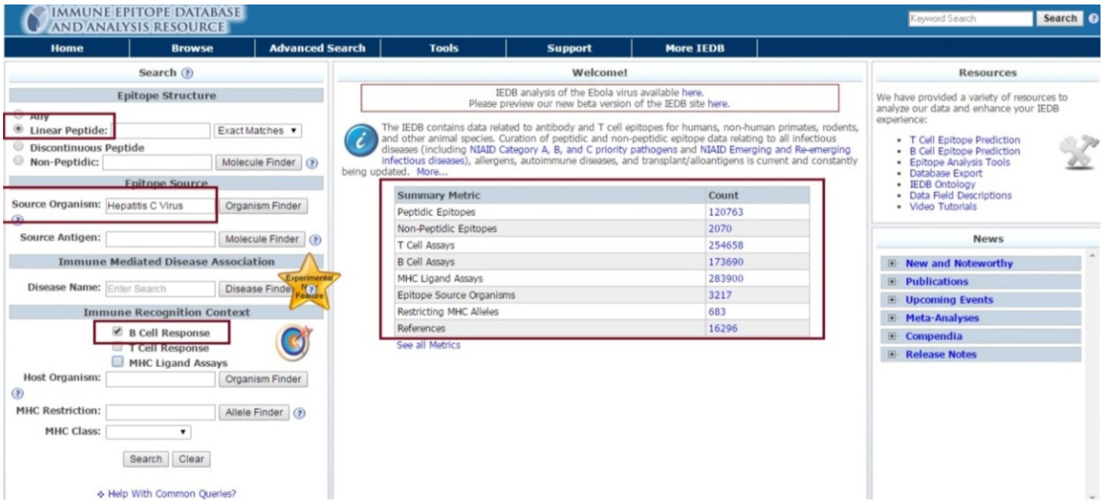

(a)

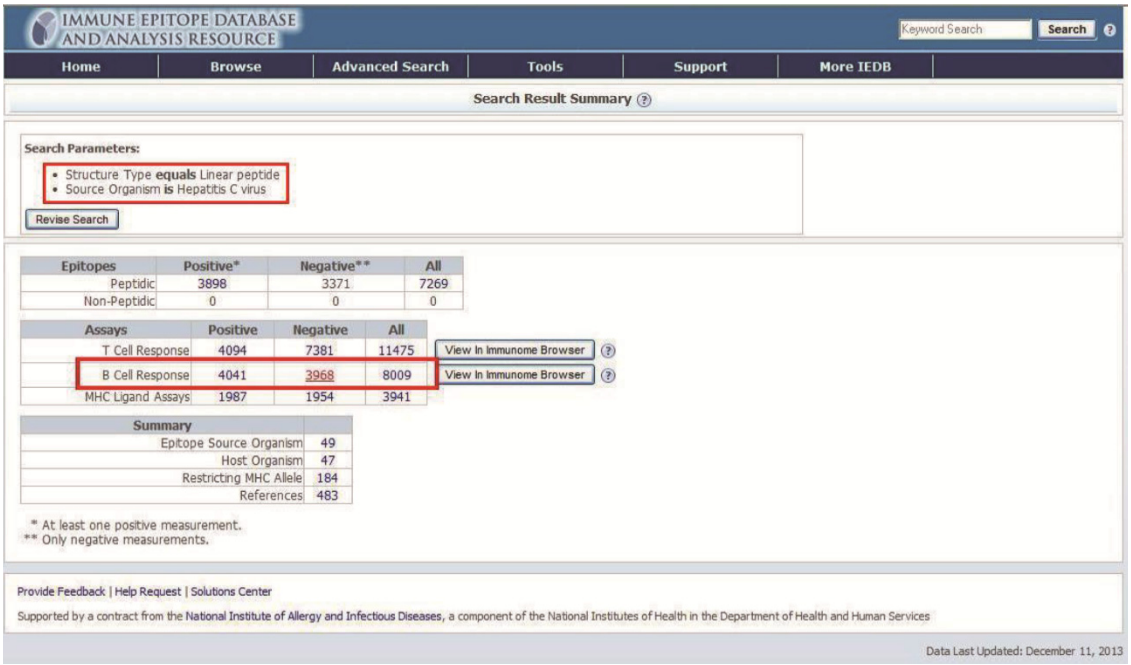

(b)

Figure S1 (a) HCV source organism. (b) A total of 8009 non-redundant linear B-cell epitopes and non B-cell epitopes are obtained
