# Supplementary material for: Prediction of linear B-cell epitopes of hepatitis C virus for vaccine development
Source: BMC Med Genomics. 2015 Dec 9;8(Suppl 4):S3. doi: 10.1186/1755-8794-8-S4-S3 (PMC4682406; doi:10.1186/1755-8794-8-S4-S3)
Supplement: Additional file 6 — Figure S2. Figure S2 Collect relevant antigenic sequences with (b) B-cell assay data, based on the (b) positive samples and (c) negative samples. [file 1755-8794-8-S4-S3-S6.pdf]

Figure S2

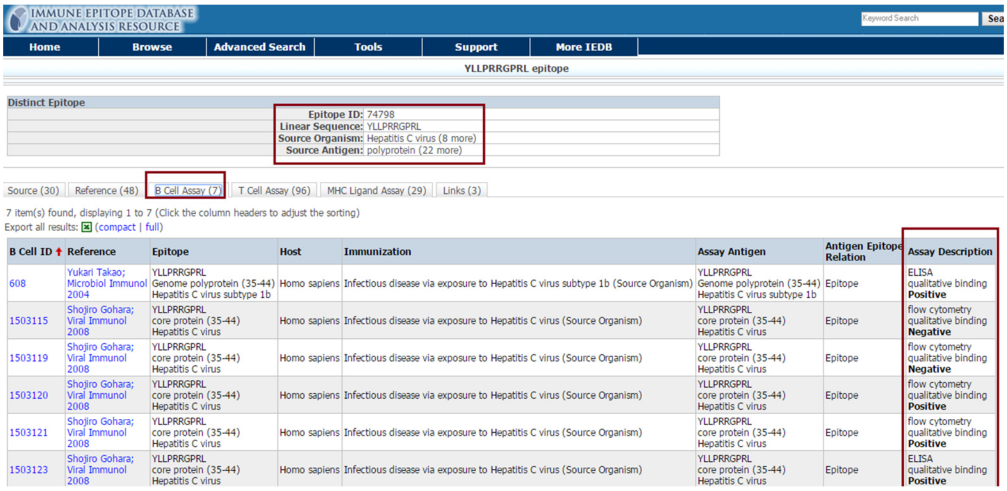

(a)

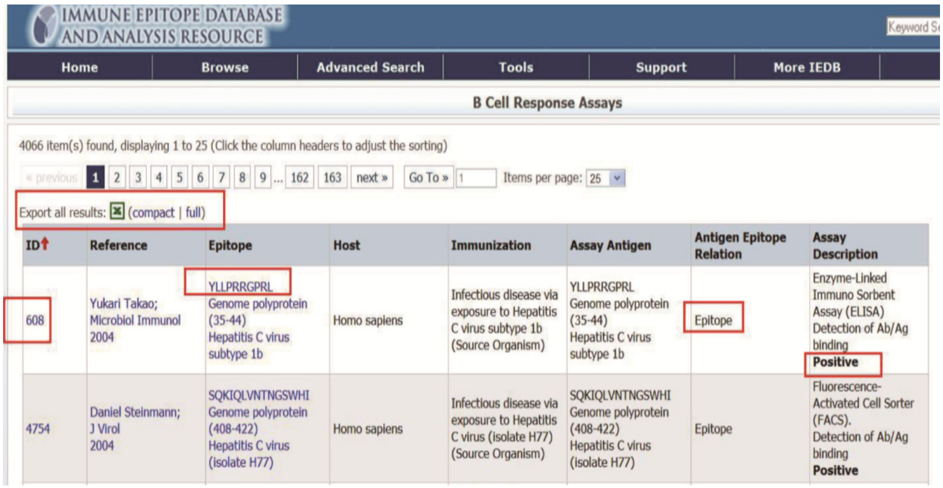

(b)

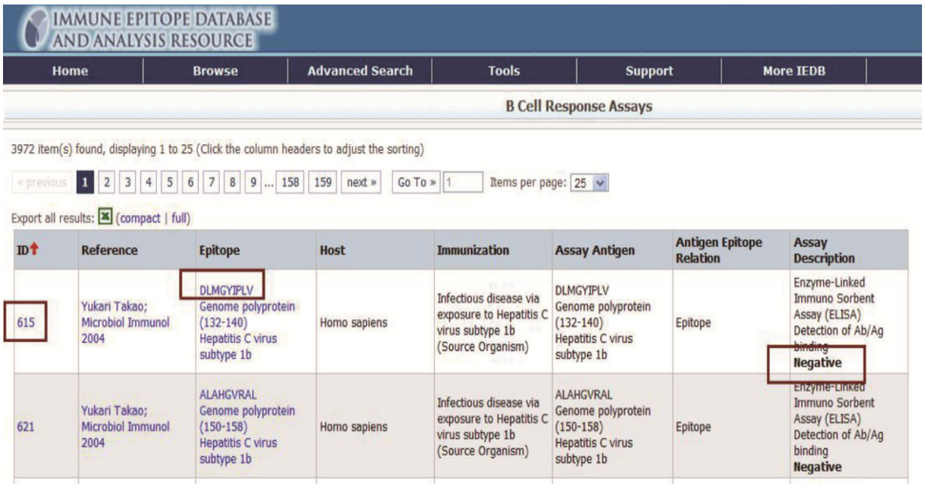

(c)

Figure S2 Collect relevant antigenic sequences with (b) B-cell assay data, based on the (b) positive samples and (c) negative samples.
