# Supplementary material for: Prediction of linear B-cell epitopes of hepatitis C virus for vaccine development
Source: BMC Med Genomics. 2015 Dec 9;8(Suppl 4):S3. doi: 10.1186/1755-8794-8-S4-S3 (PMC4682406; doi:10.1186/1755-8794-8-S4-S3)
Supplement: Additional file 7 — Table S5. The control parameters of IBCGA used. [file 1755-8794-8-S4-S3-S7.pdf]

Table S5

The used control parameters of IBCGA

Parameter	Value
Population size N_{pop}	50
Selection probability P_s	0.2
Crossover probability P_c	0.8
Mutation probability P_m	0.05
Factor number of orthogonal arrays	7
Maximum generations G_{max}	60
